# Inducing low energy availability in trained endurance male athletes results in poorer explosive power

**DOI:** 10.1007/s00421-021-04857-4

**Published:** 2021-11-26

**Authors:** Iva Jurov, Nicola Keay, Darjan Spudić, Samo Rauter

**Affiliations:** 1 Faculty of Sport, University of Ljubljana, Gortanova 22, 1000 Ljubljana, Slovenia; 2 Department of Sport and Exercise Sciences, Durham University, Durham, UK

**Keywords:** Energy availability, Performance, Endurance athletes, Cognitive restriction, Exercise metabolism, Relative energy deficiency in sport

## Abstract

**Purpose:**

Low energy availability in males needs more original research to understand its health and performance consequences. The aim of the study was to induce low energy availability in previously healthy male endurance athletes by reducing energy availability by 25% for 14 consecutive days and measure any potential changes in performance, health, mental state or energy markers.

**Methods:**

Energy availability was reduced in 12 trained, well-trained and elite endurance athletes by increasing energy expenditure and controlling energy intake. After intervention, health was assessed by blood draw, body composition was measured, energy markers by measuring resting energy expenditure, performance with three specific tests (measuring endurance, agility and explosive power) and two questionnaires were used for psychological assessment (the Three Factor Eating Questionnaire and Well-being questionnaire).

**Results:**

Reduced energy availability (22.4 ± 6.3 kcal/kg FFM/day) caused significantly lower haemoglobin values (*t*(12) = 2.652, *p* = 0.022), there was a tendency for lower iron and IGF-1 (*p* = 0.066 and *p* = 0.077, respectively). Explosive power was reduced (*t*(12) = 4.570, *p* = 0.001), lactate metabolism was altered and athletes reported poorer well-being (*t*(12) = 2.385, *p* = 0.036). Cognitive restriction was correlated with energy availability (*r* = 0.528, *p* = 0.039).

**Conclusion:**

This is the first research providing direct evidence that suboptimal energy availability negatively impacts explosive power before hormonal changes occur in male endurance athletes. It is also the first to show direct association of low energy availability and higher cognitive restriction. We also observed worse well-being and lower haemoglobin values. 25% of energy availability reduction as not enough to elicit changes in resting energy expenditure.

**Supplementary Information:**

The online version contains supplementary material available at 10.1007/s00421-021-04857-4.

## Introduction

Energy availability (EA) is important for athletes health and performance (Mountjoy et al. 2014). Assessing EA in athletes is mostly based on using simple tools (questionnaires, unconfirmed surrogate markers) that indirectly try to find connections to well-being and athletic abilities (Logue et al. 2020). There is not much original research where EA is objectively measured since all procedures represent a great burden for the athlete and the researcher (Burke et al. 2018b). Objective measurements could reveal the threshold for low energy availability (LEA), which still hasn’t been determined in male athletes. This still remains a challenge in males as clinical signs of LEA are not as apparent as in females (Mountjoy et al. 2014) Finding a threshold for LEA in men could enable us to fully understand how and when health and performance are affected (Burke et al. 2018a). Further research should focus on using objective methodology in controlled laboratory setting to enable any relevant conclusions (Logue et al. 2020). The threshold could be determined by inducing LEA in previously healthy athletes with a systematic approach in strictly monitored conditions (Jurov et al. 2021b).

The aim of this study was to reduce EA by 25% in trained, well-trained and elite endurance male athletes and observe any changes in performance, health, and well-being. This study was designed to measure EA objectively in order to avoid errors in estimations with simple tools often used in this research setting. All participants were measured in unchanged conditions first to measure their EA in normal living conditions and to confirm that they were healthy males without any pre-existing clinical signs of LEA. EA was then reduced by increasing exercise energy expenditure (EEE) and by controlling that energy intake (EI) was identical as in the normal living conditions. After the intervention we observed any changes in endurance, agility, explosive power, in blood samples, and in questionnaires assessing well-being and eating behaviours.

## Materials and methods

### Ethics approval

All participants needed to sign an informed consent before commencing all protocols for allowing data to be gathered and analysed anonymously. This research complied with the declaration of Helsinki. National medical ethical approval was acquired before the start of the study (No. 0120–202/2020/5).

### Consent to participate

Written consent was acquired from all participants prior the start of the study to participate in this study.

### Consent for publication

There is no identifying information included in this article. Written consent for publication of data for research purposes was acquired from all participants prior the start of the study.

### Study design

**Fig. 1:**
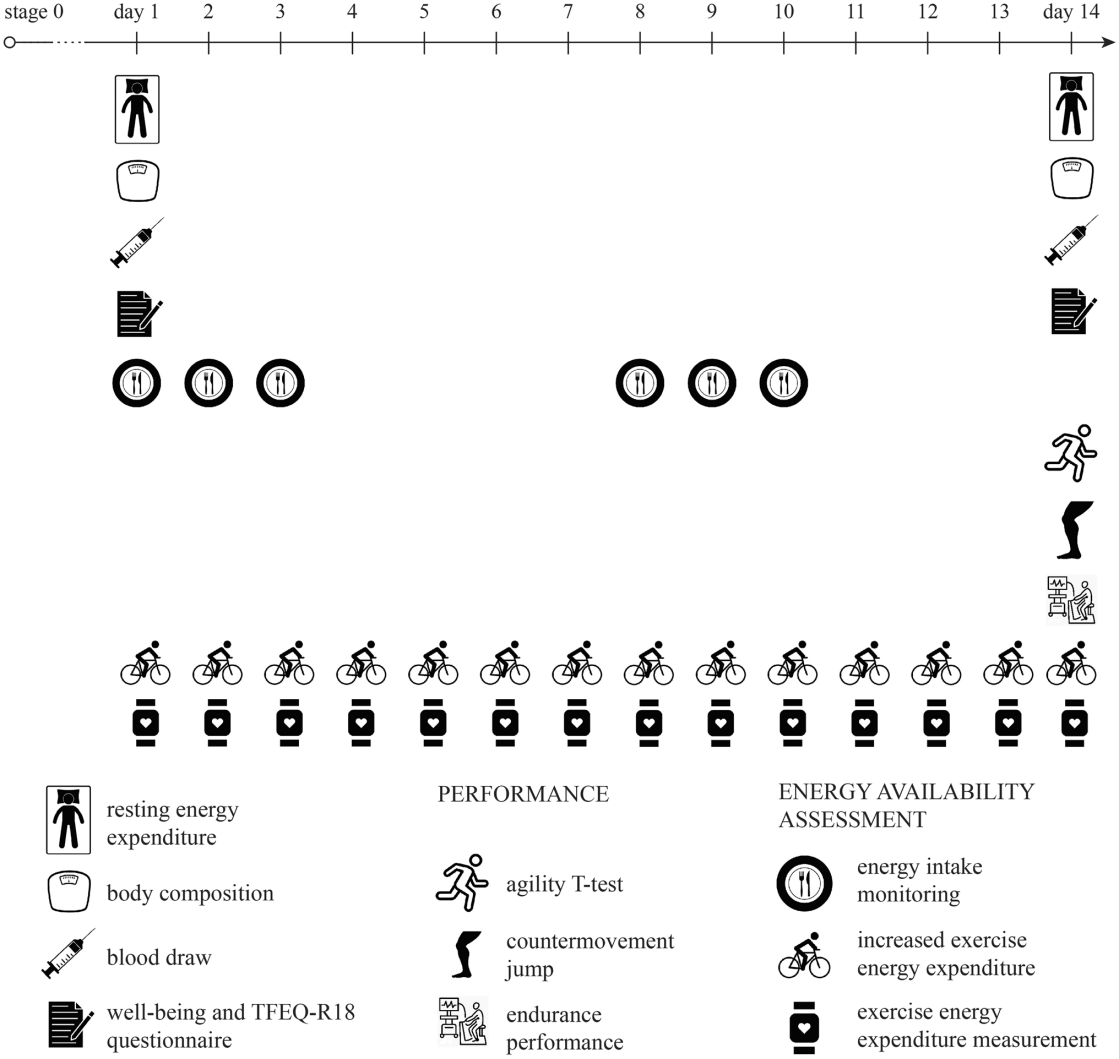
Study design

This research was set as an intervention cross-sectional controlled study. We aimed to reduce EA in trained, well-trained, and elite male endurance athletes by 25% and observe any health, performance and well-being effects. EA was measured with detailed methodology first over a 7-day long period (Jurov et al. 2021a). The main goal of this study was to reduce EA in each individual by 25% by maintaining identical dietary regime (the same EI). The EEE to ensure reduced EA was calculated and athletes had to train more under specific conditions to achieve the reduced EA values over 14 days. Finally, health, performance, and well-being measures were compared to values in each athlete under normal living conditions previously published (Jurov et al. 2021a) (Fig. 1). In this way, each athlete acted as a control and as a participant involved in the intervention.

First, athletes were assessed under normal living conditions (Jurov et al. 2021a) to monitor their baseline values and confirm inclusion criteria. In addition, energy intake (EI) and EEE were measured for seven consecutive days. Their EA in normal living conditions was calculated (stage 0 EA). Then, participants’ EA was reduced by 25% (stage 1). Between the stages there was at least a 1-month wash-out period since the measurements represent a great burden on the athlete. Before starting the intervention, we confirmed the participants had the same values by repeating vital measurements on day 1 of the intervention (Fig. 1). Finally, the results obtained after 14-days of reduced EA by 25% were compared to the values from stage 0 in each individual. All procedures took place during the competition period of the season during the period when competitions were cancelled due to coronavirus outbreak (no competitions were performed during this study).

#### EA under normal living conditions (stage 0)

A 9-day period was used to measure baseline values needed for EA calculation under normal-living conditions (the EA 0). All athletes reported EI by completing dietary diaries for 7 consecutive days. A 7-day period was used as suggested by Capling et al. stating that recording intake for 3–7 days is a reasonable compromise between scientific rigor and practicality when estimating dietary intake of athletes (Capling et al. 2017). Longer periods of EI reporting might lead to over- or underestimation. During the EI-monitoring period, EEE was monitored during all training units with heart rate monitor. Subjects were instructed to eat and train as usually. After 7 days blood samples were drawn. This was followed by 1 day of rest and then body composition was assessed and REE was measured. Finally, three performance tests were used for determining basal performance and athletes filled-in two psychological questionnaires. The details of stage 0 can be found in a recently published article (Jurov et al. 2021a).

#### 25% EA reduction stage (stage 1)

Athletes had their EA individually reduced by 25%. First, their new EA was calculated (EA 1). The EA 1 was reached by keeping their EI identical and increasing their EEE. To ensure EI was the same as in stage 0, athletes had to eat the same (identical) meals then in stage 0. Since stage 1 lasted 14 days (Fig. 1), they had to repeat their eating schedule twice (stage 0 lasted 7 days). Only water and salt intake were not limited. To ensure that participants’ EI remained the same as in stage 0, diary logs were repeated on day 1, 2, 3, and 8, 9, 10. Not completing diary logs for 14 days straight was used to reduce the burden on the athletes.

EEE was increased to reach the reduced EA values with cycling or running. Subjects exercised every day for a specific time period adjusted for their EA reduction needs. They ran or cycled at HR between 70 and 80% of their maximal HR.

To confirm that baseline values from stage 0 were present at the start of stage 1, blood was drawn, mREE, and fat free mass (FFM) were measured on day 1 (Fig. 1). Three Factor Eating Questionnaire (TFEQ-R18) and Well-being questionnaire were completed.

During the following 14 days, the 25% reduced EA state was maintained and monitored with EI and EEE measurements. On the 14th day, blood was drawn, REE and body composition was measured, performance was assessed and TFEQ-R18 and Well-being questionnaire were completed.

### Participants

**Fig. 2:**
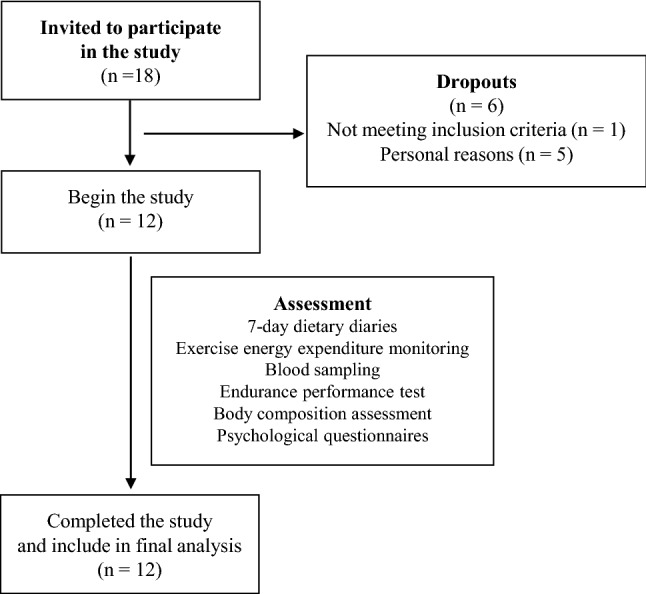
The inclusion process

Eighteen (*N* = 18) trained, well-trained and elite endurance male athletes were invited. Inclusion criteria and selection process is presented in Table 1 and Fig. 2. Twelve (*N* = 12) athletes completed all procedures.

**Table 1:** Inclusion criteria for participants

Sex	Male
Age	18–35 years
Performance level	Well trained; with VO2_max_ 55–64,9 ml/kg/min; performance level 3 or more (De Pauw et al. 2013)
BMI	1. BMI 19–25 kg/m^2^; in normal range for adult males) and body fat 5–20%
Body Fat Percentage	2. 5–20%
Health status	1. No acute disease or chronic disease in relapse (allowing only for chronic diseases that are stable and not affecting performance) 2. At the time of procedures be free of injuries and no injuries in previous three months that could affect performance
Additional criteria	3. Stable body mass for the last 12 months
	4. Not undertaking and specific diet regime
	5.At the time of procedures will refrain from alcohol consumption and any drug or other substance use
	6.Complete all procedures and report any factors that could influence changes in blood values or performance (lack of motivation due to psychological factors, factors in between measurements that could influence results etc.)

All participants needed to sign an informed consent before commencing all protocols for allowing data to be gathered and analysed anonymously.

### Subject involvement

Subjects were invited to participate in the study through national cycling and triathlon organizations, professional cycling team’s coaches. The information was also disseminated through faculty’s laboratory, where national best endurance athletes regularly perform various testings. Subjects were informed of all procedures and were selected based on inclusion criteria, high motivation and compliance.

### Procedures

#### Energy availability calculation

EA was calculated as EA = (EI-EEE)/FFM. Detailed instructions were given to all participants for weighing foods, using measuring tools and providing photographic evidence of everything ingested. Data was analyzed with Foodworks 9 Professional Edition (version 9.0.3973, Xyrix Software, Australia). EEE was measured by using wearable heart rate monitors during all exercise sessions (Polar V800, Polar Electro, Kempele, Finland).

Training load in stage 1 (EEE 1) was calculated from EA 1, which was 25% higher as EA 0 for each individual. Subjects exercised every day for a specific time period adjusted for their EA reduction needs. They ran or cycled at HR between 70 and 80% of their maximal HR.

#### Body composition assessment

Body composition was assessed using tetra polar eight-point tactile bioelectrical impedance device InBody 720 *(*Biospace, Seoul, South Korea). Participants were instructed how to be adequately hydrated prior measurements. Body composition was measured the first before all other procedures took place.

#### Resting energy expenditure assessment

REE was measured with indirect calorimetry (V2 mask (Hans Rudolph, USA), K5 (Cosmed, Albano Laziale, Rome, Italy) with Quark 8.1. PC software support) based on the Weir equation (Weir 1949; Torstveit et al. 2019), for 30 min in a thermoneutral environment, in silence, between 6.00 and 9.00 a.m, just after the body composition measurement. Participants were in fasted state, had to refrain from physical exertion in the last 12 h and any caffeine ingestion. The final 20 min were used for REE analysis (Compher et al. 2006). For predicted REE (pREE) calculation, the Harris-Benedict equation was used (Harris and Benedict 1918). The mREE/pREE ratio was then calculated to assess energy conservation.

#### Blood samples

All blood samples were drawn in the morning at 9am in a fasted state to assess complete blood count, ferritin, serum iron (Fe), triiodothyronine (T3), thyroid stimulating hormone (TSH), morning testosterone, fasting insulin, insulin like growth factor 1 (IGF-1) and 9am cortisol. Blood was collected using standard clinical procedures. Haemoglobin was analysed with Sysmex XN-550 (photometric detection, EDTA tubes), iron with Cobas c501 (colorimetric analysis, serum tubes), ZSH, T3, testosterone, cortisol and ferritin with Cobas e411 (electrochemiluminescence immunoassay, serum tubes). Serum insulin level was analyzed with a double antibody RIA (serum tubes) and for IGF-1 the RIA kit (serum tubes) was used.

#### Psychological assessment

Mental status was assessed with the TFEQ-R18 and Well-being questionnaire (Viner et al. 2015; Jurov et al. 2020b). TFEQ-R18 and its subscale, cognitive restriction, were used to look for changes in eating behaviours as used in similar research setting before. General well-being was assessed by a simple questionnaire as recommended by Hooper and Mackinnon (Hooper and Mackinnon 1995) including six subjective ratings (fatigue, sleep, stress, muscle soreness, mood and morning erections) on a 1–5 scale. The last item about morning erection was to the original set as proposed by study on professional rugby players (McLean et al. 2010) (supplementary file 1).

#### Performance testing

To test performance, three different tests were chosen to assess vertical jump height (explosive power of lower extremities), motor task execution time (agility) and maximal aerobic capacity (aerobic endurance). Performance tests were performed in exact order for each participant: general warm-up (2 min of cycling on a stationary bike at a 50 revolutions per minute at approximately 1.5 W/kg) was followed by performing mobility exercises (athletes performed arm, hip, knee and ankle mobility exercises (10 reps each); dynamic stretches of hip flexors, knee extensors, knee flexors and ankle extensors (10 reps each); and heel raise, squat, crunch resistance exercises (10 reps each).

First, countermovement jump (CMJ) test was performed using a bilateral force plate system (Type 9260AA, Kistler Instrumente AG, Winterthur, Switzerland) with Kistler MARS software (S2P Ltd., Ljubljana, Slovenia) to acquire ground reaction force. Each subject performed three to five maximal counter movement jumps before the testing. For CMJ data were sampled at 1000 Hz, filtered using a moving average filter with 50-ms window and analyzed using the built-in module for CMJ. Test execution was supervised from the experienced researcher to improve proficiency in jumping technique [24]. Before each jump, participants were instructed to stand up straight and still on the center of the force plate with their hands akimbo. This hand position remained the same during the entire movement. From this position, participants initiated a fast downward movement until a crouching position with a knee angle of about 90°, followed by a jump for maximal height as quickly and explosively as possible. Three valid trials were performed with one-minute recovery period. The main outcome measure was CMJ height in centimeters that was calculated from the maximum velocity [25].

To asses motor task execution time, validated modified agility t-test was used, as described by Haj-Sassi, et al. (2011). The *T*-test was performed after 5 min rest from vertical jump testing without additional warm up. Each participant had three trials (each trail consisting of two sprints separated by 30 s rest). The rest between trials was 90 s. Cones were placed in a T-shape layout, 5 and 2.5 m apart, respectively. Subjects were instructed to sprint and change direction as fast as possible. They began with both feet 0.3 m behind the starting line (A). At their own discretion subject sprinted forward to cone B and touched the base of it with the right hand. Facing forward and without crossing feet, they shuffled to the left to cone C and touched its base with the left hand. Subjects then shuffled to the right to cone D and touched its base with the right hand. They shuffled back to the left to cone B and touched its base. Finally, subjects ran backward as quickly as possible to cross the finish line (cone A) ending the first sprint. The second sprint started after 30 s rest. Three such trials separated by 90 s rest were performed, all with verbal encouragement. The subject who crossed one foot in to each direction (firstly starting with shuffling to the left) front of the other, failed to touch the base of the cone or failed to face forward throughout, had to repeat the test. The time to complete each repetition was measured using one pair of the electronic timing system sensors (Witty Timing System, Microgate, Bolzano) mounted on tripods. They were set approximately 0.75 m above the floor positioned 2 m apart facing each other on either side of the starting line (A). The time of best repetition (seconds) and three repetition average were used in further analysis.

After 1 h of rest, endurance was measured with the incremental test to exhaustion. Heart rate, ventilatory, and gas data were collected during the incremental test with metabolic cart (K5, Cosmed, Italy). All measurements were performed in the physiological laboratory with ambient temperature of 21 °C. For measuring VO2max, the following procedure was performed. After a 15-min warm-up on bicycle set up on a cycle ergometer (Cyclus 2, Leipzig, Germany), workload constantly increased until volitional exhaustion (100 W + 20 W every minute). Lactate was analyzed in capillary blood drop from the earlobe. Samples were obtained at rest before any physical activities, at the end of the test and 5 min after the test. Lactate was analyzed with the blood lactate analyzer Biosen C_Line (EKF Diagnostics, Germany).

### Data analysis

For statistical analysis, SPSS was used with a significance level of < 0.05 (version 25.0, IBM SPSS Statistics, Chicago, Illinois, USA). The normality of distribution for each dependent variable was explored during statistical analysis. Paired samples T-test was used to analyse changes in health, performance and metabolic factors in stages 0 and 1. Descriptive statistics was used to present anthropological factors, age, energy parameters, blood values, performance and psychological evaluation in all stages. Pearson’s correlation coefficient was used to find associations between EA and other measured parameters.

Minimal sample size needed for this study (*N* = 12) was calculated with the G*power 3.1.9.2 software, assuming expected effect size 0,47 for EA as reported in previous studies (Heikura et al. 2018), with type I(a) error = 0,05 and 30% dropout of participants.

This research complied with the declaration of Helsinki. National medical ethical approval was acquired before the start of the study (No. 0120–202/2020/5).

## Results

**Fig. 3:**
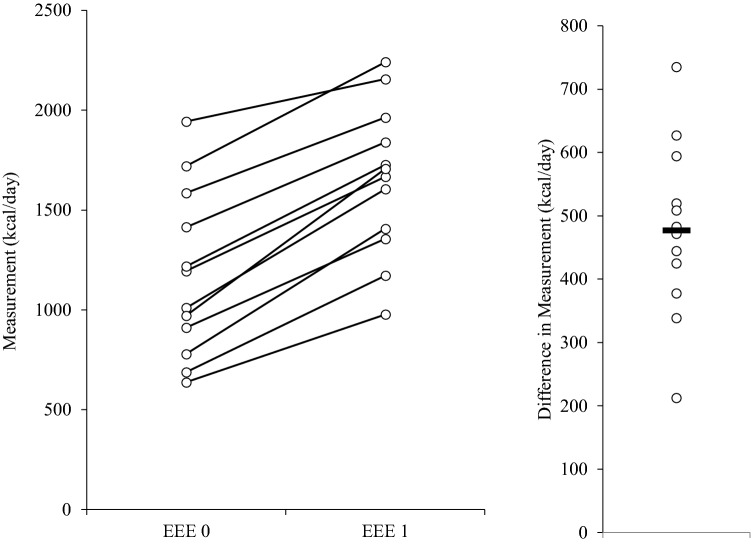
The exercise energy expenditure (EEE) increase from normal living conditions (stage 0) to 25% EA reduction intervention (stage 1)

**Fig. 4:**
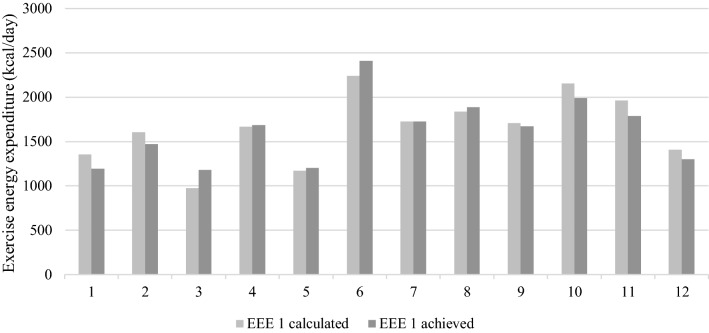
Subjects were able to achieve exercise energy expenditure that was calculated for EA reduction very well

**Fig. 5:**
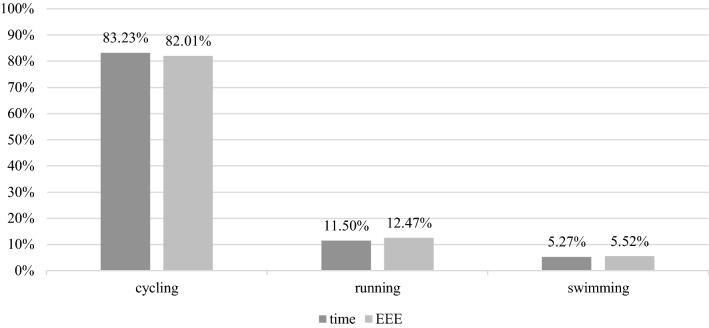
Time and exercise energy expenditure (EEE) divided into training modalities as measured in stage 1

The physiological parameters of athletes involved in this study can be found in Supplementary file 1, Table 6. We confirmed all subject were trained endurance athletes (cyclists and triathletes). Body mass, FFM and body fat were lower after the intervention, but the change was not significant. The biggest tendency was for lower body fat (*t*(12) = 1.805, *p* = 0.099, *r* = 0.48). On average, EEE 1 was 470.3 ± 134.2 kcal higher than EEE 0 (Fig. 3). The achieved EEE 1 was 1.5% lower compared to calculated EEE 1 (Fig. 4). Training modalities were closely related between the stages (Fig. 5).

On weekly average, participants spent 15 h 24 min cycling, 1 h 41 min running and 45 min swimming. Average daily training time was 2 h 33 min per day. Other.

### Energy parameters

We found no significant intervention effect in mREE or mREE/pREE. 3/12 subjects showed mREE/pREE < 0.9 (Supplementary file 1, Table 5). Mean EA in stage 1 was 22.4 ± 6.3 kcal/kg FFM/day.

### Blood values

Differences in blood parameters in stage 1 were significant in haemoglobin and there was a tendency for change in iron values (Table 2). There was also a tendency for lower IGF-1 after the intervention (*p* = 0.077). In general, all parameters were lower at the end of the stage, except for testosterone values. There were no significant correlations with the achieved EA in stage 1.

**Table 2:** T-test comparing blood parameters before and after intervention

Marker	Reference range	Mean 1a	Mean 1b	Mean Difference	Std. Deviation	Std. Error Mean	95% CI Lower	95% CI Upper	*t*	*df*	sig. (2-tailed)
Haemoglobin (g/L)	138—175	149.58	146.00	3.58	4.68	1.35	0.61	6.56	2.652	11	.022*
S-Iron (μmol/L)	5.8—34.5	23.91	19.12	4.79	8.04	2.32	− 0.31	9.90	2.065	11	**.063**
S-TSH (mIU/L)	0.27—4.20	2.40	2.01	0.39	1.02	0.30	− 0.26	1.04	1.318	11	.214
S-T3 (pmol/L)	3.1—6.8	4.74	4.60	0.14	0.54	0.16	− 0.20	0.48	0.892	11	.391
S-Testosterone (nmol/L)	8.64—29.0	19.46	20.22	− 0.76	6.68	1.93	− 5.01	3.48	− 0.396	11	.700
S-cortisol (nmol/L)	166.0–507.0	414.16	428.21	− 14.05	91.58	26.44	− 72.24	44.14	− 0.531	11	.606
S-ferritin (μg/L)	30—400	123.19	120.39	2.80	21.28	6.14	− 10.72	16.32	0.456	11	.657
Insulin (mE/L)	2–29.1	3.79	3.08	0.71	2.32	0.67	− 0.77	2.18	1.057	11	.313
IGF-1 (μg/L)	83.4–232.7	201.50	177.08	24.42	43.29	12.50	− 3.09	51.92	1.954	11	.077
IGF-1 SD		0.059	− 0.370	0.429	0.772	0.223	− 0.061	0.919	1.926	11	.080

^*^significance set at *p* < 0.05

### Performance

We observed statistically significant differences in [La]_max_ and countermovement jump before and after intervention in stage 1 (Table 3). Except for the *T*-test (*r* = 0.548, *p* = 0.032), performance parameters were not correlated with achieved EA in stage.

**Table 3:** Paired samples test of performance related parameters before (stage 0) and after the intervention (stage 1)

	Mean	Std. Deviation	Std. Error Mean	95% CI Lower	95% CI Upper	*t*	*df*	sig. (2-tailed)
VO_2max_ 0—1 (ml/min/kg)	− 0.37	6.92	2.00	− 4.76	4.03	− .184	11	.858
PO_0—1 (W)	− 2.50	18.15	5.24	− 14.03	9.03	− .477	11	.643
RPO_0—1 (W/kg)	− 0.07	0.28	0.08	− 0.25	0.11	− .856	11	.410
AT_0—1 (ml/min/kg)	− 3.20	8.00	2.31	− 8.28	1.88	− 1.386	11	.193
RC_0—1 (ml/min/kg)	− 3.81	8.78	2.54	− 9.39	1.77	− 1.502	11	.161
[La]_max__0—1 (mmol/l)	2.02	2.46	0.71	0.45	3.59	2.839	11	.016*
[La]_5 min_ _0—1 (mmol/l)	1.22	2.46	0.71	− 0.34	2.79	1.722	11	.113
T-test_0—1 (s)	0.11	0.39	0.11	− 0.13	0.36	1.017	11	.331
CMJ_0—1 (m)	0.030	0.022	0.006	0.015	0.044	4.570	11	.001*

^*^Statistical significance set at *p* < .05

(*VO*_*2max*_ maximal oxygen consumption, *PO*  peak power output, *RPO* relative power output, *AT*  anaerobic threshold, *RC* respiratory compensation point, *[La]*_*max*_ lactate concentration at the end of the test, *[La]*_*5min*_ lactate concentration 5 min after the end of the test, *CMJ*  countermovement jump)

### Psychological

Average values of TFEQ and cognitive restriction were not significantly different at the end of stage 1. Well-being was significantly lower after the intervention (*t*(12) = 2.385, *p* = 0.036, *r* = 0.58), had significantly lower value (Supplementary file 1, Table 5). There was a tendency for higher cognitive restriction after lowered EA.

EA was associated with cognitive restriction in stage 1—if EA was lower, cognitive restriction was higher (*r* = 0.528, *p* = 0.039).

## Discussion

Inducing LEA by 25% in stage 1 resulted in mean EA 22.4 ± 6.3 kcal/kg FFM/day. On average, it took subjects two and a half hours of monitored exercise per day to reach the desired EEE. We found performance changes, poorer well-being and lower haemoglobin. After 14 days of EA reduction by 25 we did not observe any changes in hormones or energy markers.

The intervention resulted in lower body mass, FFM and fat, but the differences were not big enough to be statistically significant. The biggest difference was seen in body fat (*t*(12) = 1.805, *p* = 0.099)), which was a desirable outcome for the participants’ optimal body composition.

### Energy conservation

Lower EA did not result in changes of mREE or mREE/pREE. There are two possible mechanisms to explain this. The first is that 14-days was not enough to cause energy conservation as detected by mREE/pREE. The time duration needed for mREE/pREE change is not known when EA is lowered by 25%. Woods et al. (2018) conducted research on trained cyclists that was not the same intervention study design, but it was similar. They increased training load at 120% in week 1 and then at 140% in week 2 and 150% in week 3. This resulted in significant mREE decrease. In addition, Woods et al. (2017) also subjected elite rowers to 4 weeks of intensified training load, which also resulted in mREE decrease. We might speculate that longer duration of 3 weeks in our study could result in significant mREE (and thus mREE/pREE) differences. The second possible mechanism is that in our study design, increased EEE could cause the EPOC effect that would influence measurement of REE. Subjects had to refrain from physical activity 12 h before each REE measurement, which could be too little since their training units were significantly increased (Jurov et al. 2020a). However, we observed that after the intervention 3 subjects had the mREE/pREE < 0.9, which is a critical value indicating energy conservation (De Souza et al. 2007). Only one subject showed energy conservation prior to intervention. More research is needed to confirm if mREE/pREE could be used as an assessment tool for LEA in men.

### Changes in blood values

Haemoglobin values dropped significantly. Since haemoglobin influences VO_2max_, this effect is undesirable in terms of aerobic performance. A prolonged LEA lasting 21 weeks in 24.6 kcal/kg FFM/day resulted in immunosuppression, including dysregulated haematopoiesis, that was shown by downregulation of genes responsible for haematopoiesis regulation (Sarin et al. 2019) in female bodybuilders. Also, a tendency for lower iron in our sample is consistent with knowledge that low iron can be caused by inadequate EI (Castell et al. 2019). Although these studies are in line with our findings, their study design is not identical to our research. The possibility that haemoglobin can be reduced by LEA needs further investigation. We would like to suggest that potential longer duration of lowered EA of 25% could lead to poorer endurance performance because of suboptimal oxygen-binding capacity.

Although other parameters did not show statistically significant changes, we observed that this intervention caused a drop in all blood parameters with the exception of testosterone. De Souza et al. (2019) concluded that EA must be below 30 kcal/kg FFM/day in men for any alterations in reproductive function. Although mean EA was lower than 30 in this study, it seems that this stage was not intense or long enough to cause lower mean testosterone values. In addition, testosterone function (morning erections) was not different at the end of this study. This is in accordance with currently the only 2 studies which modulated EA by reducing EI or increasing EEE that also failed to show any endocrine changes in 4 and 5 days long interventions (Koehler et al. 2016; Papageorgiou et al. 2017).

### Performance

After the intervention we observed reduced explosive power and altered lactate metabolism. Participants reached lower lactate values at the end of the incremental test. This could be explained by lower glycogen reserves in our participants who had lower EA during 14 days. We did not measure glycogen reserves directly, but this mechanism has been proposed before in a similar research setting (Jurov et al. 2020a). Furthermore, it is clear that vertical jump performance as a measure of lower extremity explosive power has decreased significantly between 1.5 cm and 4.4 cm (p = 0.001), indicating additionally that LEA could influence high intensity performance first.

### Psychological changes

Looking into psychological evaluation, the well-being questionnaire detected changes in EA. At the end of the intervention subjects reported poorer well-being status. Higher cognitive restriction was associated with poorer EA, which is a novel finding. Our finding is in line with a study by Gibbs et al. (2013) that showed LEA in exercising women had increased cognitive restriction. However, their methods for determining LEA were not objective. To the best of our knowledge, this paper is the first to provide direct association of EA and cognitive restriction. Our intervention confirmed that it indeed seems that men are resilient to LEA induced hormonal alterations. Fortunately, screening for changes of well-being and cognitive restriction could be helpful for early detection. What is more, reduced explosive power and impaired lactate metabolism in our research suggest the direct negative performance effects of LEA.

### Limitations

The authors would like to acknowledge that although all participants are trained endurance athletes, some of them fall into the well-trained and also elite group according to De Pauw et al. (2013). Finding sufficiently large sample size of only elite athletes is a challenge in such a research setting. This is why we used athletes from all three performance groups. To our knowledge, this is the biggest sample in a controlled setting for measuring EA in trained endurance male athletes and can thus provide some relevant conclusions for scientists, athletes, their coaches and other sports practitioners. Only this approach can enable further exploration of LEA effects on the male hypothalamic-pituitary–gonadal axis (Logue et al. 2020) and performance consequences.

## Conclusions

This is the first research providing direct evidence that suboptimal EA negatively impacts explosive power before hormonal changes occur in male endurance athletes. It is also the first to show direct association of reduced EA and higher cognitive restriction. Lowering EA by 25% caused lower haemoglobin values. We also observed a tendency for lower body fat values. It seems that suboptimal performance in explosive power occurs sooner than hormonal changes in trained endurance athletes with lowered EA. Longer duration of lowered EA for 25% could result in poorer endurance performance due to reduced oxygen-binding capacity. We suggest future research should aim to lower EA even more severely in a similar manner, which might cause other hormonal changes that could be part of LEA assessment tool in future. Using the Well-being questionnaire and measuring cognitive restriction at intervals across the season could aid in preventing the negative performance effects of reduced EA. Evaluation of well-being could be especially useful when training blocks change during the periodization plan, as changes in the training schedule can result in reduced EA. Finally, the goal for finding the threshold for LEA can be accomplished by further limiting EA in similar laboratory conditions and looking for additional hormonal changes.

## Supplementary Information

Below is the link to the electronic supplementary material.

Supplementary file1 (DOCX 21 KB)

## Abbreviations

[La]_5 min_: Lactate concentration 5 min after the end of the test
[La]_max_: Lactate concentration at the end of the test
AT: Anaerobic threshold
CMJ: Countermovement jump
CR: Cognitive restriction
EA: Energy availability
EEE: Exercise energy expenditure
EI: Energy intake
Fe: Serum iron
FFM: Fat free mass
IGF-1: Insulin-like growth factor 1
LEA: Low energy availability
mREE: Measured resting energy expenditure
PO: Peak power output
pREE: Predicted resting energy expenditure
RC: Respiratory compensation point
RED-S: Relative energy deficiency in sport syndrome
RPO: Relative power output
T3: Triiodothyronine
TFEQ-R18: Three factor eating questionnaire
TSH: Thyroid stimulating hormone
VO_2max_: Maximal oxygen uptake

## DeclarationsDeclarations

### Data availability

The datasets generated during and/or analysed during the current study are available from the corresponding author on reasonable request.
