# Supplementary material for: Inducing low energy availability in trained endurance male athletes results in poorer explosive power
Source: Eur J Appl Physiol. 2021 Nov 26;122(2):503–13. doi: 10.1007/s00421-021-04857-4 (PMC8617370; doi:10.1007/s00421-021-04857-4)
Supplement: Supplementary file 1 — Supplementary file1 (DOCX 21 KB) [file 421_2021_4857_MOESM1_ESM.docx]

**Supplementary file 1**

*Table 4: Well-being subjective questionnaire with scoring system*

|  | 5 | 4 | 3 | 2 | 1 |
| --- | --- | --- | --- | --- | --- |
| FATIGUE | very fresh | fresh | normal | more tired than normal | always tired |
| SLEEP QUALITY | very restful | good | difficulty falling asleep | restless sleep | insomnia |
| GENERAL MUSCLE SORENESS | feeling great | feeling good | normal | increase in soreness/tightness | very sore |
| STRESS LEVELS | very relaxed | relaxed | normal | feeling stressed | highly stressed |
| MOOD | very positive mood | a generally good mood | less interested in others &/or activities than usual | snappiness at teammates, family and co-workers | highly annoyed/ irritable/ down |
| MORNING ERECTIONS | >5 per week |  |  | <2 per week | almost never/not present |

*Table 5: Paired samples test of energy parameters and well-being evaluation prior and after 14 days of reduced energy availability by 25%*

|  | Mean | Std. Deviation | Std. Error Mean | 95% CI Lower | 95% CI Upper | t | df | sig. (2-tailed) |
| --- | --- | --- | --- | --- | --- | --- | --- | --- |
| EEE (kcal/day) | -470.312 | 134.186 | 37.217 | -551.400 | -389.224 | -12.637 | 11 | 0.000 |
| REE (kcal/day) | -129.917 | 411.705 | 118.849 | -391.502 | 131.668 | -1.093 | 11 | 0.298 |
| mREE/pREE | -0.075 | 0.239 | 0.069 | -0.226 | 0.077 | -1.086 | 11 | 0.301 |
| WB | 2.6 | 3.8 | 1.1 | 0.2 | 5.0 | 2.385 | 11 | 0.036 |
| TFEQ-R18 | 0.9 | 8.2 | 2.4 | -4.3 | 6.1 | 0.386 | 11 | 0.707 |

(EEE = exercise energy expenditure, mREE = measured resting energy expenditure, pREE = predicted resting energy expenditure, TFEQ-R18 = Three Factor Eating Questionnaire, WB = well-being questionnaire)

*Table 6: Anthropometric data and performance related parameters of athletes included in the study*

|  | Minimum | Maximum | Mean | SD |
| --- | --- | --- | --- | --- |
| age (years) | 19 | 35 | 27.5 | 5.7 |
| BH_0 (cm) | 170.5 | 187.5 | 179.8 | 4.4 |
| BM_0 (kg) | 64.8 | 77.0 | 71.8 | 3.6 |
| FFM_0 (kg) | 59.0 | 69.4 | 64.5 | 3.7 |
| FFM_0 (%) | 85.9% | 94.0% | 89.8% | 2.5% |
| FAT_0 (%) | 6.0% | 14.0% | 10.2% | 2.5% |
| VO_2max__0 (ml/min/kg) | 56.80 | 76.10 | 67.49 | 6.74 |
| PO_0 (W) | 350.00 | 470.00 | 402.50 | 40.03 |
| RPO_0 (W/kg) | 4.90 | 6.28 | 5.60 | 0.47 |
| AT_0 (ml/min/kg) | 38.20 | 55.30 | 47.10 | 5.99 |
| RC_0 (ml/min/kg) | 46.00 | 67.70 | 57.48 | 7.12 |
| [La]_max__0 (mmol/l) | 6.44 | 15.52 | 10.80 | 2.46 |
| [La]_5min__0 (mmol/l) | 8.60 | 16.45 | 11.29 | 2.07 |
| T-test_0 (s) | 5.71 | 7.03 | 6.49 | 0.40 |
| CMJ_0 (m) | 0.24 | 0.43 | 0.32 | 0.05 |

(BH=body height, BM=body mass, FFM=fat-free mass, FAT=fat mass, VO_2max_=maximal oxygen consumption, PO=peak power output, RPO=relative power output, AT=anaerobic threshold, RC=respiratory compensation point, [La]_max_=lactate concentration at the end of the test, [La]_5min_=lactate concentration 5 minutes after the end of the test, CMJ=countermovement jump)
